# A System of Practical Medicine by American Authors

**Published:** 1898-06

**Authors:** 


**A System of Practical Medicine by American Authors.** Edited by ALFRED LEE LOOMIS, M.D., and WILLIAM GILMAN THOMPSON, M.D. Vol. II.—*Diseases of the Respiratory System—Diseases of the Circulatory System and the Mediastinum—Diseases of the Blood—Diseases of the Kidneys—Diseases of the Bladder and Prostate Gland.* Pp. 5—11, 19—941. London: Henry Kimpton. 1897.

In the opening portion of this volume Dr. S. Edwin Solly gives an outline of the diseases of the nose, naso-pharynx, and larynx. As regards tuberculosis of the larynx, he points out that "the danger of tuberculous laryngitis lies not so much in the difficulty in curing the local disease, as in the fact that it is almost invariably allied with a tendency to the general dissemination of tubercle with an absence of self-limitation;" he believes that in nearly all instances it is a secondary infection from a thoracic deposit, and that the infiltration takes place as a rule in the submucous tissues, ulceration occurring subsequently. He states that "early recognition and appropriate climatic change, with local treatment, have a very marked influence on the prognosis," but he has not thought it worth while to mention any general medicinal treatment.

Dr. Elbridge G. Cutler writes on the physical signs of pulmonary disease, hæmoptysis, physical signs of cardiac disease, cardiac atrophy, cardiac failure, acute and chronic myocarditis, and the fat heart. We are a little surprised to find it said that "auscultatory percussion has been advised for determining the cardiac boundaries, but it does not seem to present any advantages over the ordinary methods, and is not, therefore, recommended to the general practitioner." We do not discover any especial mention of Sansom's pleximeter, which certainly does give a more accurate means of estimating the area of heart-dulness than can be obtained by the finger, nor do we find any mention of the use of the Röntgen rays for estimating the varying sizes of the heart under different conditions: we hope that a future edition of this volume will give us more modern and improved methods for estimating the effects of treatment on the size of the heart. Schott has found that the fluorescent screen may be made to give important information, and the combined auscultation-percussion method has been found to be fairly trustworthy.

Dr. Herbert B. Whitney writes on non-tubercular diseases of the pleura, and he comments fully on the fact that at least forty per cent. of all individuals with sero-fibrinous effusion will eventually develop tuberculosis, and further that the results of inoculation and *post-mortem* examination confirm the belief that even a larger proportion of such pleurisies are of genuinely tubercular character, The presence in a purulent effusion of certain micro-organisms differentiates the sero-fibrinous from the purulent pleurisies, and these latter are grouped in accordance with the individual special organisms, as follows:—(1) Streptococcus pyogenes; (2) The pneumococcus of Fränkel; (3) The bacillus tuberculosis; (4) Saprophytic germs.

The section on the examination of the blood is the joint work of Dr. F. C. Shattuck and Dr. Richard C. Cabot, and is well up to date inasmuch as it is the work of pioneers in this field. The use of Daland's hæmatocrit is said to diminish greatly the tedium of counting the corpuscles, inasmuch as with this centrifuge "a count is usually done in two minutes." The specific gravity method as devised by Roy and modified by Lloyd Jones does not appear to have been in favour, as it is not mentioned by the authors: we have found this method to be of comparatively easy application and quite as trustworthy or more so than the hæmacytometer of Gowers. The different varieties of leucocytes are indicated by the Ehrlich-Biondi method, the triple stain bringing out the characteristic points, whilst the erythrocytes are stained yellow. This article is illustrated by a series of coloured plates, somewhat resembling those which are to be found in Dr. Cabot's excellent monograph.

The first portion of diseases of the kidneys is by Dr. Henry P. Loomis, and it comprises nephritis, with amyloid degeneration, and renal hyperæmia. He defines Bright's disease as a bilateral hæmatogenous non-suppurative nephritis, and states that all nephritis can be divided into two great divisions—acute and chronic diffuse nephritis, the former generally ending in recovery, the latter always in death—the former being comparatively rare, the latter very frequent. "The writer has been especially impressed with the fact that a very large proportion of the patients who give no renal symptoms yet have albumin and casts in the urine," and "it is the exception to find kidneys in persons over forty years of age which will show *under the microscope* even a relatively normal structure."

An outline of uræmia, by Dr. Thomas D. Coleman, brings this interesting volume to a close; and we hope that the Editor will be able to continue the good work already done, and that volumes iii. and iv. will quickly follow the excellent volumes which have already been issued.

